# Management of Complex Diverticular Disease With Fistulation Into an Ovarian Dermoid Cyst

**DOI:** 10.7759/cureus.95924

**Published:** 2025-11-01

**Authors:** Jamie Cransberg, Amelia L Davis, Genevieve Gibbons

**Affiliations:** 1 General Surgery, Western Australian Country Health Service, Bunbury, AUS; 2 General Surgery, Sir Charles Gairdner Hospital, Perth, AUS; 3 General Surgery, Western Australian Country Health Service, Albany, AUS

**Keywords:** complicated diverticular disease, dermoid ovarian cyst, dermoid teratoma, diverticular fistula, general and colorectal surgeon, rural surgery

## Abstract

Diverticular disease is prevalent amongst the adult Australian population. Diverticular disease can be complicated by fistula formation, most commonly involving the bladder or bowel. We report a rare case of an immunocompromised 69-year-old woman with a diverticular fistula extending into a complex right ovarian dermoid cyst. The patient presented to and was surgically managed in a rural Western Australian hospital with an emergency laparotomy, en bloc resection, and Hartmann’s procedure. The patient recovered well and is awaiting elective colostomy reversal. This case demonstrates a rare complication of diverticular disease leading to a delayed presentation and demonstrates that timely surgical intervention can enable optimal outcomes despite geographic isolation.

## Introduction

Diverticular disease, characterised by thin-walled outpouchings known as diverticula of the bowel, is common, affecting approximately one-third of Australians over the age of 45 years [1]. Diverticula can intermittently become inflamed, causing a condition known as diverticulitis. Uncomplicated diverticulitis is often successfully managed with antibiotics, though it may recur throughout a patient’s lifetime. Diverticulitis can become complicated due to intramural or extramural abscesses, perforation, or fistulation into adjacent structures [1].

Diverticular fistulas most commonly form with the bladder or small bowel [1,2]. Surgical management is often required in cases of diverticular disease with fistula formation. Rarely, diverticular disease can be complicated by fistula formation into ovarian structures, including tubo-ovarian abscesses and ovarian cysts [3-5]. We describe a rare case of diverticular disease with fistulation to an ovarian dermoid cyst, requiring emergent surgical management in a rural Australian hospital.

The hospital of initial presentation is a public hospital in rural Western Australia, over 400 kilometres from the nearest tertiary hospital. The region serviced by this hospital is classified as MM3 (large rural town) by the Modified Monash Model [6].

## Case presentation

A 69-year-old female presented twice to the emergency department of a rural hospital with a history of fever and three weeks of intermittent lower abdominal pain, which was improving. The patient had a background of known diverticular disease with multiple previous episodes of diverticulitis and immunosuppression on adalimumab for rheumatoid arthritis. Interestingly, the patient had been experiencing intermittent abdominal pain for the past 18 months, with computed tomography (CT) imaging showing lymphadenopathy retroperitoneally and in the mesentery. This was further investigated for lymphoma and found to be photopenic on positron emission tomography imaging with unremarkable haematological indices for haemoglobin, lymphocyte, neutrophil, and platelet counts. Multidisciplinary discussion resulted in surveillance of lymphadenopathy. Socially, the patient is a cattle farmer who was living independently prior to admission [7].

**Figure 1:**
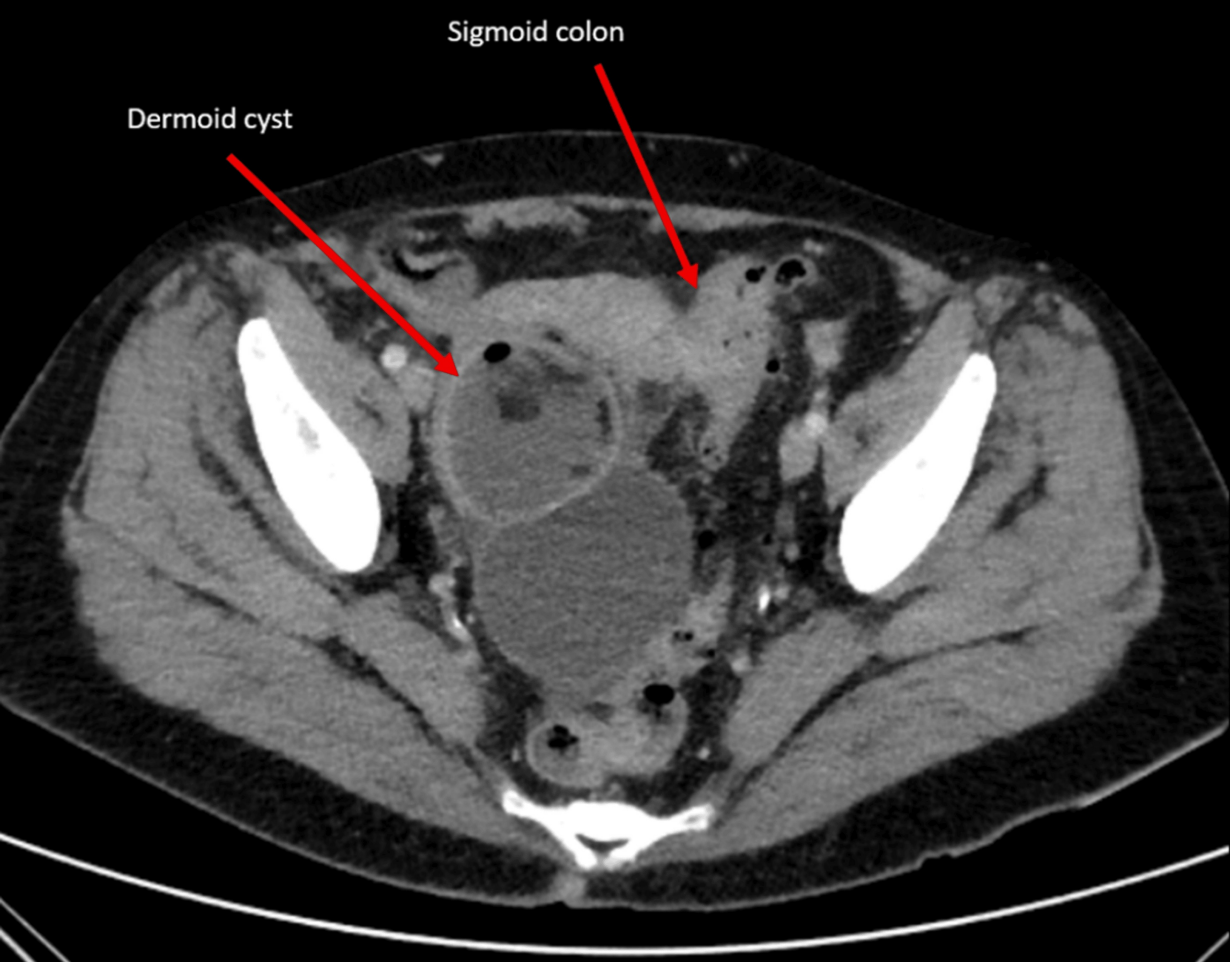
Axial portal venous contrast computed tomography scan demonstrating an inflamed sigmoid colon and fistulous communication with large multiloculate dermoid cysts (red arrows). Gas locules can be seen within the dermoid cyst.

On the first presentation, history revealed a recent cough and abdominal pain. A temperature of 38.4°C was recorded in the emergency department, with a relative tachycardia of 90 beats per minute. An abdominal examination revealed tenderness in the lower abdomen. Pathology results demonstrated a normal white cell count of 9.04 x 10^9^/L, with an elevated C-reactive protein of 193 mg/L (Table 1). Renal function and liver function tests were unremarkable. Urinalysis was negative. A chest radiograph showed clear lung fields, and respiratory swabs were negative for COVID, influenza, and respiratory syncytial virus (RSV). A decision was made to treat for a possible atypical chest infection with oral antibiotics with close general practitioner review. Blood cultures returned positive for gram-negative bacilli, and the patient was instructed to present to the emergency department for assessment. CT imaging of the abdomen at this time demonstrated active diverticulitis of the sigmoid colon with fistulation into the dermoid cyst, which contained locules of gas not present on imaging two months prior (Figure 1). Tumour markers, including carcinoembryonic antigen (CEA), cancer antigen 19-9 (CA 19-9), and cancer antigen 125 (CA 125), were all within normal limits (Table 1).

**Table 1:** Laboratory results from blood tests taken on the day of admission. CA 125: cancer antigen 125; CEA: carcinoembryonic antigen.

Blood test	Result	Normal range (units)
Haemoglobin	99	115-160 (g/L)
White cell count	9.4	4.0-11.0 (10^9^/L)
Platelet count	434	150-400 (10^9^/L)
Neutrophils	5.09	2.0-7.5 (10^9^/L)
C-reactive protein	170	<5.0 (mg/L)
Lipase	88	<220 (U/L)
Lactate	0.5	0.5-2.0 (mmol/L)
CA 125	13	<35 (U/mL)
CEA	1	<5 (µg/L)

Management

**Figure 2:**
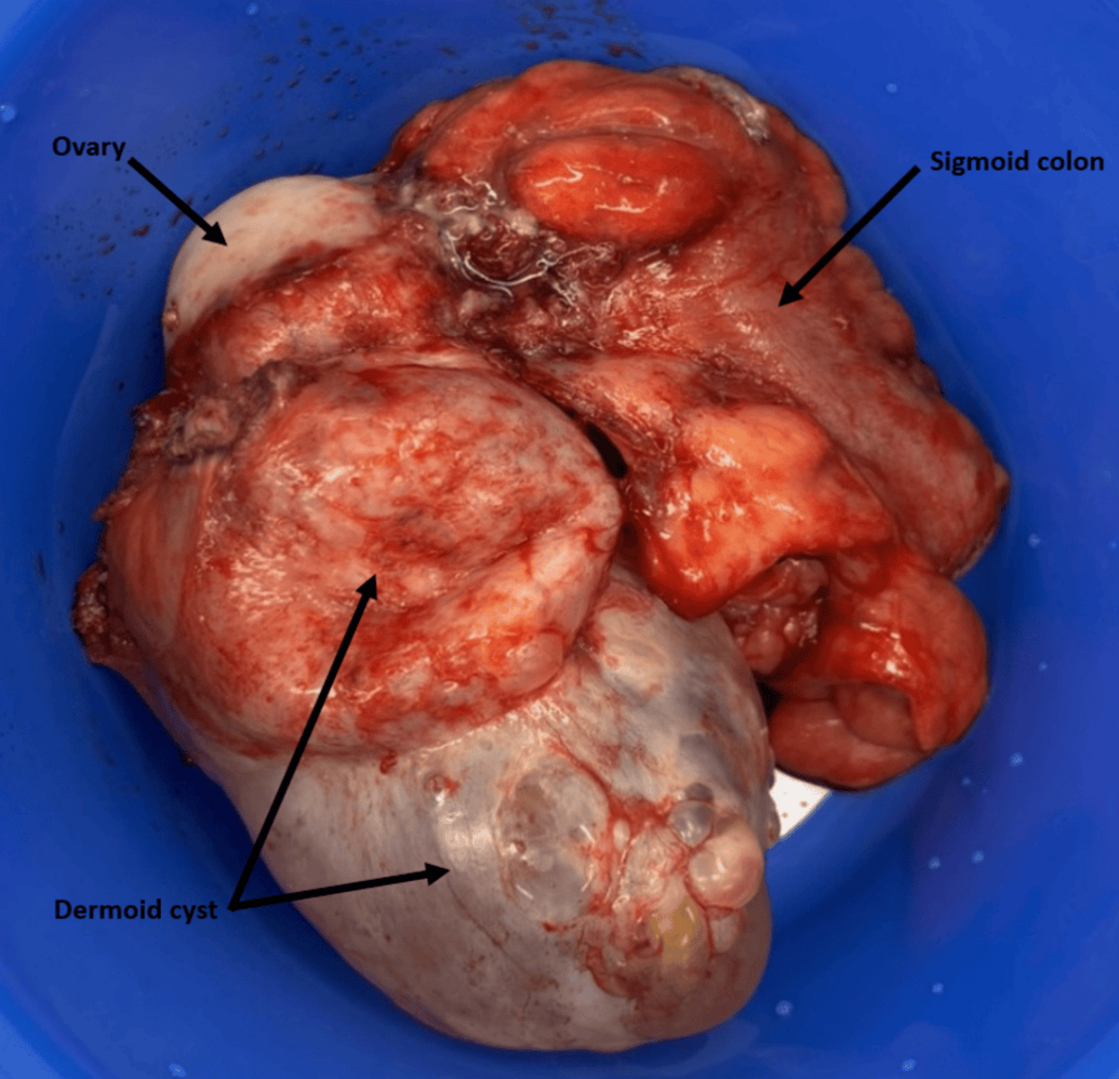
Clinical photograph of en bloc resection of the sigmoid colon, dermoid cyst, right ovary, and right fallopian tube with annotations.

**Figure 3:**
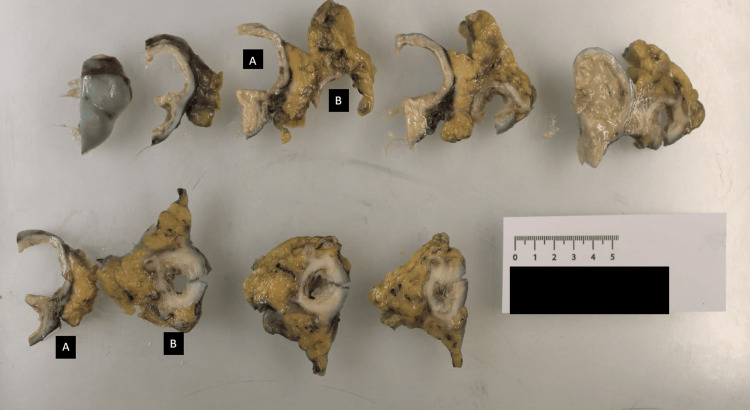
Macroscopic pathology images of the specimen were prepared in transverse slices. The dermoid cyst (A) and sigmoid colon (B) are shown.

The patient was admitted to the hospital under the care of a rural general surgeon with subspecialty colorectal surgery training. Initially, the patient was stabilised with fluid resuscitation and antibiotics. Within 72 hours of admission, the patient underwent a laparotomy with en bloc resection of the dermoid cyst, fallopian tube, and the diseased sigmoid colon. Intraoperative findings correlated well with preoperative imaging, revealing a complex dermoid cyst adherent to an inflamed sigmoid colon, with associated fistula (Figures 2, 3). Due to significant intra-abdominal inflammation, primary bowel anastomosis was deemed unviable, and the decision was made to form a rectal stump and end colostomy, known as a Hartman’s procedure.

Outcome

**Figure 4:**
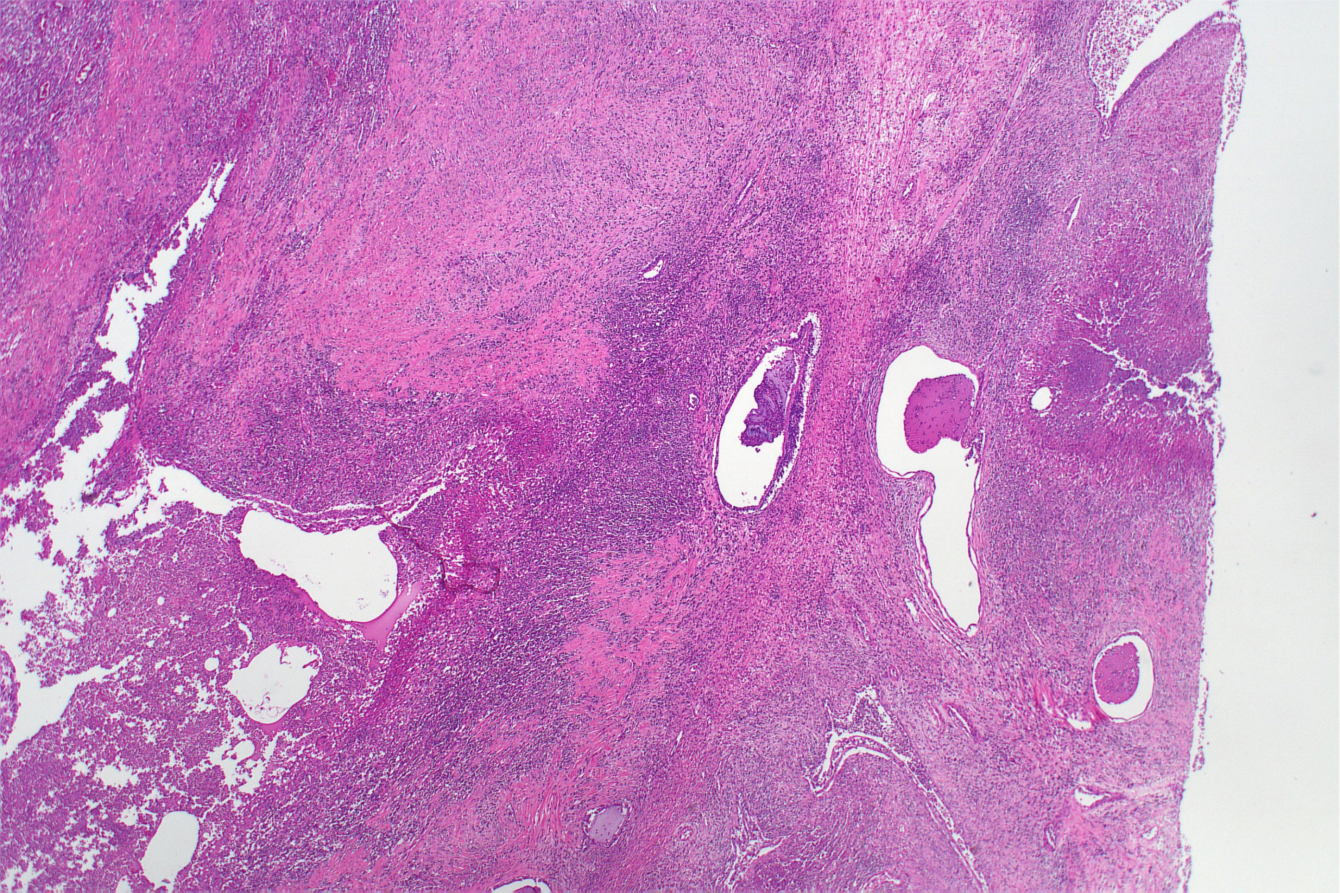
Histopathology slide demonstrating an inflamed cystic teratoma with focal respiratory epithelial cysts. The specimen has been haematoxylin and eosin-stained and is viewed at 2x power.

Intravenous antibiotics ceased after significant clinical and biochemical improvement postoperatively. The colostomy was active after 48 hours, and the patient and her family received education from a specialised stoma nurse prior to discharge. After a nine-day admission, the patient was discharged home to her usual place of residence. Histopathology results confirmed an inflamed colon and a mature cystic teratoma with no evidence of malignancy (Figure 4). Following discharge, the patient experienced parastomal inflammation requiring close monitoring via a specialist stoma nurse. Within three months postoperatively, the patient was deemed a candidate for reversal of Hartmann’s and waitlisted for an elective theatre date. A postoperative CT showed only mild residual para-aortic lymphadenopathy, indicating that this may have been a reactive phenomenon since the onset of symptoms 18 months prior.

## Discussion

A rare occurrence

It is well recognised that diverticular fistulas are unlikely to spontaneously close. Because of this, both local and international guidelines recommend consideration of surgery for diverticular disease complicated by fistula [1,2]. Principles of fistula management include controlling sepsis, optimising nutritional status, and defining the anatomy of the fistula before proceeding to definitive surgical management [8]. Ideally, surgical management of complicated diverticular disease is delayed until after the acute episode has subsided; however, in cases where the patient is systemically unwell, such as described in this case report, emergency surgery may be indicated.

Cases of fistulation between dermoid cysts and colonic diverticular disease are exceedingly rare in the literature. There are case reports of both benign and malignant dermoid cysts invading into the bowel in the absence of diverticular disease [4,5]. Other ovarian cancers also have been reported to fistulate with bowel in the presence or absence of diverticular disease, highlighting the importance of considering malignancy in preoperative workup. There are multiple reports of diverticular fistulation into simple ovarian cysts [9]. Quintela et al. described two cases of diverticular fistulation into simple ovarian cysts managed initially with radiologically guided drainage, followed by staged en bloc resection [8]. Fistulation of diverticular disease with tubo-ovarian abscess has been described in the literature, also with successful management with en bloc resection [3]. Management and outcomes described in our case report are in line with those described in cases of fistulation with other tubo-ovarian structures. Interestingly, the patient had been found to have mesenteric and retroperitoneal lymphadenopathy 12 months prior; however, the possibility of a diverticular fistula with the complex ovarian cyst was not considered. Increased awareness of this phenomenon may have led to more rapid diagnosis and management in an elective setting.

Colorectal surgery in rural Australia

Western Australia’s geographically dispersed population creates unique challenges in healthcare delivery, particularly specialist care [10,11]. Rural general surgeons in Australia care for a larger number of patients per surgeon compared with their metropolitan counterparts. Scarcity of surgical services can create barriers to care for rural patients [12]. Despite limited access in some regions, evidence suggests that well-resourced rural areas experience equivalent outcomes in colorectal surgery compared with metropolitan counterparts [13]. The site of this case report was staffed with a general surgeon with subspecialty colorectal surgery training and a specialist stoma nurse. The case report demonstrates that complex colorectal surgery can be provided in a timely and optimal manner in a rural setting.

## Conclusions

This case demonstrates a rare complication of diverticular disease, with fistula formation between sigmoid diverticulitis and a complex ovarian dermoid cyst. Surgical management of diverticular fistulae is well recognised and is ideally completed as an elective operation. In this case, emergency surgical management was deemed most suitable due to uncontrolled infection. As a result of prompt surgical management, the patient made an excellent recovery. Additionally, the case is evidence that complex cases of diverticular disease can be operatively managed in a rural setting with appropriately trained specialist surgeons and multidisciplinary care in place.
